# A Comprehensive Review: Bovine Respiratory Disease, Current Insights into Epidemiology, Diagnostic Challenges, and Vaccination

**DOI:** 10.3390/vetsci12080778

**Published:** 2025-08-20

**Authors:** Stephanie O’Donoghue, Sinéad M. Waters, Derek W. Morris, Bernadette Earley

**Affiliations:** 1 Animal and Bioscience Research Department, Animal and Grassland Research and Innovation Centre (AGRIC), Teagasc, Grange, Dunsany, C15 PW93 Co. Meath, Ireland; 2 School of Biological and Chemical Sciences, University of Galway, H91 TK33 Galway, Ireland; sinead.waters@universityofgalway.ie (S.M.W.); derek.morris@universityofgalway.ie (D.W.M.)

**Keywords:** bovine respiratory disease (BRD), diagnosis, clinical respiratory scores, behaviour, thoracic ultrasonography (TUS), bacterial pathogens, viral pathogens, vaccination

## Abstract

The aim of this comprehensive review is to synthesize current knowledge on bovine respiratory disease (BRD), enhance diagnostic strategies, and support effective prevention and management practises. BRD remains a leading cause of morbidity and mortality in cattle, driven by a complex interplay of viral and bacterial pathogens, host factors, environmental stressors, and management conditions. Its prevalence (2.1% to 20.2%) varies across geographical regions, age groups, and diagnostic methods. BRD leads to significant economic losses through direct impacts such as mortality, reduced growth rates, and lighter carcass weights, as well as indirect costs like market restrictions and long-term productivity declines. Diagnosing BRD is challenging due to its non-specific clinical signs and frequent subclinical presentations. Traditional diagnostic tools like clinical respiratory scoring (CRS) systems provide structure but suffer from low sensitivity and subjectivity. Behavioural monitoring shows promise by detecting early changes in feeding, movement, and social behaviours. Thoracic auscultation is widely used but limited in accuracy. Thoracic ultrasonography (TUS) stands out as a more sensitive method for detecting subclinical disease and correlating with growth outcomes. Combining CRS with TUS enhances early and accurate detection. Advancing diagnostic approaches is critical for improving animal health and minimizing economic losses in cattle production systems.

## Simple Summary

Bovine Respiratory Disease (BRD) is a major health issue in cattle worldwide, caused by a mix of pathogens, host factors, and environmental stresses. Diagnosis is difficult due to variable and often subtle symptoms. Common methods include clinical respiratory scoring (CRS), behavioural monitoring, auscultation, and thoracic ultrasonography (TUS). TUS is especially effective at detecting subclinical cases and predicting poor growth. BRD prevalence varies by region, age, and season, with higher rates in winter. Economically, BRD leads to mortality, reduced growth, and lower carcass value, causing significant direct and indirect losses. Early detection combining CRS and TUS improves outcomes and reduces costs.

## 1. Introduction

Bovine respiratory disease (BRD), also known as pneumonia, is a multifactorial condition that affects cattle across all age groups [1,2,3,4,5,6,7,8,9,10]. It is the leading cause of mortality in pre-weaned dairy calves [11,12]. The disease arises from a complex interplay of viral and bacterial pathogens, host-related factors, environmental stressors, and management practises [13,14,15]. The term BRD is commonly used to encompass both upper (URT) and lower respiratory tract (LRT) illnesses in cattle, which frequently involve mixed infections of viral and bacterial origin [16]. The crises associated with BRD are multifaceted. Clinically, it compromises animal welfare, hinders growth rates, and can result in chronic pulmonary damage. From an economic standpoint, BRD is one of the most costly diseases in cattle production systems, contributing to losses due to treatment expenses, reduced weight gain, increased culling rates, and mortality. Estimates suggest that BRD costs the U.S. beef industry alone over USD 1 billion annually in direct and indirect losses [17,18,19]. Similar burdens are reported globally, affecting both intensive and extensive farming operations. Despite decades of research, BRD remains a persistent challenge due to its complex etiology and the variability in diagnostic criteria, prevention strategies, and treatment responses. A deeper understanding of the disease dynamics, including pathogen–host–environment interactions, is critical for developing effective interventions. Moreover, antimicrobial resistance associated with BRD pathogens adds urgency to adopting evidence-based management and therapeutic strategies [20]. The objective of this comprehensive review is to provide an overview of the existing literature on bovine respiratory BRD, with a particular emphasis on clinical diagnostics as well as evaluating the complex interplay of infectious agents, host factors, stressors, and management practises that contribute to its onset and progression.

## 2. Materials and Methods

A comprehensive literature review was conducted to identify relevant studies addressing bovine respiratory disease (BRD) in cattle. Multiple electronic databases were searched, including Web of Science, PubMed, Science Citation Index Expanded (SCI-Expanded), Scopus, and CAB Abstracts (CABI). Additional searches were performed via Google Scholar to capture grey literature and articles not indexed in the primary databases. A wide range of keywords and search phrases were employed using Boolean operators (AND, OR) to ensure both breadth and specificity. The search focused on six thematic areas: disease-related terms (e.g., “bovine respiratory disease”, “BRD complex”, “respiratory tract infections in cattle”, “cattle pneumonia”, “BRD pathogens”, “respiratory co-infections”, “viral and bacterial synergy”); diagnosis and clinical signs (e.g., “clinical respiratory scoring”, “respiratory scoring systems”, “rectal temperature in BRD”, “visual assessment of cattle”, “clinical signs of BRD”); animal behaviour (e.g., “behavioural changes in sick cattle”, “depression scores in BRD”, “feeding behaviour and BRD”, “locomotion and respiratory disease”); epidemiology and prevalence (e.g., “BRD prevalence”, “incidence of respiratory disease in feedlot cattle”, “risk factors for BRD”, “BRD outbreaks”, “seasonal variation in BRD”); economic aspects (e.g., “economic impact of BRD”, “cost of treatment for BRD”, “losses due to BRD”, “feedlot economics and respiratory disease”, “economic burden of BRD in beef/dairy industry”); and treatment and prevention (e.g., “antimicrobial treatment for BRD”, “vaccination against BRD”, “antimicrobial resistance in BRD pathogens”, “BRD metaphylaxis”, “preventive strategies for respiratory infections”). Search filters were applied to prioritize literature published within the last 10 to 15 years; however, high-impact foundational studies published earlier were also included when deemed scientifically relevant to contextualize current findings. Following the application of filters, the search identified a total of 181 studies. Articles were included if they: focused on BRD in cattle (beef or dairy); reported primary data or were systematic/narrative reviews relevant to the six thematic areas; were published in English. The extracted data included: study design (e.g., observational, experimental, clinical trial, review); population (animal) characteristics (e.g., cattle breed, age, production system); definitions and diagnostic criteria for BRD; identified pathogens (viral and/or bacterial); interventions or treatment strategies, including vaccinations; outcome measures (e.g., morbidity, mortality, economic impact). Studies were assessed using the following criteria: risk of bias, consistency of results, directness of evidence, precision, and publication bias. Based on these criteria, the quality of evidence was categorized as high, moderate, low, or very low. In cases where conflicting results were identified among studies, findings were analyzed in the context of their methodology, diagnostic criteria and study population.

## 3. Economic Impact of BRD

The health of livestock and productivity levels are intrinsically linked. Animal diseases pose a threat to animal welfare, the environment, public health and the economy [17]. Animal diseases pose significant threats to animal welfare, the environment, public health, and the economy [17]. The costs associated with animal disease outbreaks can be categorized as direct or indirect [17]. Direct costs include visible losses such as animal deaths, reduced production yields, and slower growth rates, as well as less obvious impacts like decreased fertility leading to fewer offspring or restricted market access due to disease presence [18]. Indirect costs are those that occur in other markets (international trade, tourism) after the declaration of disease absence [17]. Often disease outbreaks can lead to reduced productivity and performance throughout the animal’s lifetime [18].

In Australia, a study evaluated the economic impact of BRD in Australian feedlot cattle reported that animals exhibiting subclinical and clinical BRD had carcass weights reduced by 16 kg and 24.1 kg, respectively, and generated returns that were AUD 67.10 (EUR 37.40) and AUD 213.90 (EUR 119.23) lower compared to healthy animals [19]. Additionally, mortality among early fetal and neonatal calves significantly contributes to elevated production costs [20].

An US national survey [21] reported that the median annual costs for medicine and labour to treat pre-weaned calves for BRD were USD 11.00 (EUR 9.42) and USD 15.00 (EUR 12.84)/affected calf, respectively. Adjusted mean annual BRD vaccine cost for pre-weaned calves (USD 7.67 (EUR 6.57)/animal) was significantly greater than that for cows (USD 3.18 (EUR 2.72)/animal) and heifers (USD 4.48 (EUR 3.84)/animal) while vaccination costs varied by age and ranged from USD 2.25 (EUR 1.93) to USD 6.25 (EUR 5.35)/head. In the US, using longitudinal treatment data from a large cohort of 11,470 pre-weaned dairy calves in California it was estimated that the average short-term economic cost of BRD was USD 42.15 (EUR 36.08) per affected calf [22]. This estimate included expenses such as treatment costs, including the use of anti-inflammatory medications [22]. Another study, across the US [23] reported that of 104,100 dairy replacement heifers from across the US, 36.6% had one or more cases diagnosed within the first 120 days of age with the highest risk of new cases occurring prior to weaning. Comparison of the raising cost for heifers with BRD and those without a recorded history of BRD resulted in an estimated cost per incident occurring in the first 120 days of age of USD 252 (EUR 215.66) or USD 282 (EUR 241.33). Efforts to treat these infections continue to rely largely on the use antimicrobial drugs, which through overuse, specifically in the agricultural sector, can lead to antimicrobial resistance (AMR) [24]. A further Australian study estimated that cattle that received three or more antibiotic treatments for BRD generated, on average, (covering treatment, reduced growth, and raising costs) AUD 384.97 (EUR 214.55) less in returns compared to untreated animals [19].

A study in the Netherlands [25] quantified the economic impact of BRD on veal calves, by measuring losses in hot carcass weight (HCW) and carcass quality. Calves experiencing one BRD episode lost on average 8 kg of HCW, resulting in financial losses ranging from EUR 36.8 to EUR 64.8 depending on the breed (Belgian Holstein Friesian (BHF), Red Holstein Friesian (RHF), crossbreds, or Belgian Blue (BB)). In veal calves with three BRD episodes, particularly BB and BHF, losses were much higher. BRD also increased the odds of inferior carcass quality and undesirable meat colour, further compounding financial losses. In France, a modelling study [26] suggested that eliminating BRD in beef calves could boost national beef-sector productivity by ~5.1%, equivalent to EUR 95.5 million annually in revenue and would have a much lower effect on the dairy systems (dairy calves, dairy young bulls or veal calves). In summary, BRD imposes substantial direct economic costs (treatment, mortality, weight losses) and indirect costs (impaired fertility, degraded carcass quality, trade limitations). Addressing both treatment costs and productivity losses is essential to mitigate the disease’s multifaceted effects, thereby improving animal health outcomes, optimizing production efficiency, and enhancing the overall sustainability of livestock industries worldwide. Furthermore, there is a critical need for more comprehensive economic modelling that integrates short-term treatment costs with long-term productivity losses across different production systems and regions. Such models would improve the accuracy of economic impact assessments and inform targeted resource allocation to enhance disease management and industry sustainability globally.

## 4. Prevalence of BRD

BRD is present among cattle populations worldwide. The prevalence of BRD varies widely across regions, seasons, and production systems. This variation can be attributed to a complex interplay of environmental, management, animal-specific, and diagnostic factors. Cold, damp weather and high stocking densities increase stress and facilitate the spread of respiratory pathogens. Poor ventilation and management practises, including inadequate colostrum intake or vaccination, also contribute to higher disease rates. Additionally, differences in diagnostic methods, such as clinical scoring versus thoracic ultrasonography, significantly affect reported prevalence. Production systems (e.g., intensive indoor vs. pasture-based) further influence exposure risk and disease outcomes. In Ireland, BRD was the largest cause of mortality in calves aged between one and six months of age in 2023 [27]. The estimated prevalence of BRD in Irish spring calving dairy herds was reported to be 4% [28], which is lower or like those found in other dairy production systems. A large-scale study of BRD on dairy farms in California found a BRD prevalence of 8.2% based on calves scoring positive by observed clinical signs [22]. A separate study, which did not use thoracic auscultation or TUS, but included a larger sample of dairies, estimated a 6.9% prevalence of BRD in 4636 calves across 100 dairies in California [29]. A BRD prevalence of 14% was reported in calves in Wisconsin during the winter months, based on assessments using the Wisconsin respiratory scoring system [30]. In a separate study, herd-level BRD prevalence on dairy farms in Quebec, identified by lung consolidation of ≥3 cm on thoracic ultrasonography (TUS), was recorded at 8% in the summer and rose to 15% in the winter [3]. In France, the farm level incidence of acute BRD was 9.8%, with the cumulative incidence at animal level at 2.1% [31].

A Belgian study reported that 20.2% of calves exhibited a CRS of 5 or higher, while 16.1% of calves had a TUS of 6 cm or greater [32]. In Swiss veal calves, the prevalence of key BRD viruses were evaluated from nasal swabs with findings showing a prevalence of 2.1%, 3.3%, 53.5%, 4.1% and 0% for bovine respiratory syncytial virus (BRSV), bovine parainfluenza virus type 3 (BPI3V), bovine coronavirus (BCoV), influenza D virus (IDV), and influenza C virus (ICV), respectively [8]. The wide variation in BRD prevalence is mainly driven by differences in several factors including: Climate: colder, wetter conditions promote pathogen survival and cattle stress. Herd density and management: overcrowding, poor ventilation, and inadequate preventive measures increase risk. Diagnostic criteria: variation in how BRD is detected (clinical signs vs. ultrasound) causes differences in reported prevalence. Production systems: indoor, intensive systems generally show higher prevalence than pasture-based systems. These factors highlight the need for regionally tailored prevention and control strategies that consider local climate, management practises, and diagnostic methods. In summary, diagnostic approaches, including clinical scoring systems and TUS, strongly influence reported prevalence. These findings highlight the need for standardized diagnostic criteria and support the importance of targeted prevention strategies and surveillance tailored to regional risk factors. Additionally, the marked seasonal and management-related variations in BRD prevalence suggest that prevention and treatment plans must be tailored to local risk factors rather than applying a one-size-fits-all approach.

## 5. Key Pathogens Implicated in BRD Pathogenesis

Although not the only mechanism of BRD infection, it is believed that initial viral infections compromise the respiratory tract epithelium making it more susceptible to bacterial colonization and subsequently leading to secondary bacterial infections [33,34]. The often polymicrobial nature of BRD can impact disease severity, with certain combinations of pathogens resulting in enhanced disease [15]. Although BRD affects cattle of all ages, it is often more severe in younger calves [35]. In 2023, one to six months old calves (n = 285) accounted for 41% of all cases submitted to the Irish national veterinary diagnostic labs [27]. For example, out of the 285 calves that were submitted for post-mortem BRD diagnostics, the distribution of identified agents was as follows: bacterial infections (73.3%), parasitic infections (12.6%), viral infections (10.2%), and cases with no identifiable agent represented 3.9% of the total submissions. There are a range of viral and bacterial agents that are often implicated in BRD cases, many of which are commensals within the bovine respiratory tract [36]. Due to the multifactorial nature of disease onset and the interaction between the various viral and bacterial pathogens, BRD is difficult to control and prevent [37,38]. Although single-pathogen infections can initiate outbreaks of BRD, co-infections—most commonly involving a primary viral infection that disrupts the URT microbiota and facilitates secondary bacterial colonization of the LRT—are the more prevalent pathogenic mechanism. The following sections of this review will detail the main viral and bacterial pathogens commonly associated with BRD, highlighting their roles in disease progression and interactions within the respiratory tract.

### 5.1. Viral Agents Associated with BRD

Common viral pathogens include bovine herpes virus-1 (BoHV-1), BRSV, BPIV-3, bovine viral diarrhoea virus (BVDV), BCoV, bovine adenovirus (BAV) and bovine rhinitis A and B [39]. Other emerging viruses associated with BRD include Influenza D (IDV) [39,40], Influenza C (ICV) [41] and ungulate tetraparvovirus 1 (UTPV1) [39,42]. BRD is driven by multiple viral agents, often acting in combination, which exacerbates disease severity. An overview of the characteristics, mechanisms of host entry and immunosuppression of several of the most implicated viruses in BRD is provided in the following sections and in Table 1. 

### 5.2. Bovine Herpesvirus 1 (BoHV-1)

In 2015, on Irish dairy farms, a bulk milk seroprevalence of 80% for BoHV-1 was reported [43]. More recently, a detailed analysis of 4361 Irish dairy herds with complete data from 2018 to 2024 revealed consistently high BoHV-1 prevalence [44]. In 2024, 83% of all herds tested positive at least once, with annual rates ranging from 82% to 90%. Of the 7839 herds studied, 66.6% were vaccinated or began vaccination during the period, with coverage rising from 69% in 2018 to 73% in 2024 [44]. Prevalence among unvaccinated herds increased from 68.6% to 78.8%, while it remained consistently high in vaccinated herds. The proportion of unvaccinated, BoHV-1-negative herds declined significantly, from 10% in 2018 to 4% in 2024, highlighting increased infection rates in this group [44]. Additionally, a herd-level BoHV-1 prevalence of 90% was reported in beef suckler cow herds, indicating that BoHV-1 is likely endemic in the Irish cattle population [45]. Acute infection with BoHV-1 is usually initiated in the mucosal epithelium and can lead to high levels of virus shedding, making it highly contagious [46]. BoHV-1 can establish latency within its host [46], which often results in reactivation and subsequent acute infections. During BoHV-1 initial infection, the virus encounters receptors of the local sensory nerves, where it attaches and penetrates the nerve cell in the nasal mucosa [47]. BoHV-1 usually remains latent within the ganglionic neurons of cattle that are present within the peripheral nervous system [46] and can reactivate during times of stress and immunosuppression.

### 5.3. Bovine Respiratory Syncytial Virus (BRSV)

BRSV is commonly associated with respiratory infection in cattle [48] and shares similar patterns of pathogenesis to its human counterpart HRSV [49]. A study investigating the presence of BRD pathogens in recently weaned cattle in Ireland found BRSV to be one of the most frequently detected viral pathogens, present in 16% of calves tested [2]. Furthermore, BRSV was identified as one of the most detected pathogens, with a prevalence of 11.6% in nasal swab samples collected from Irish calves aged three months or younger [40].

### 5.4. Bovine Parainfluenza 3 (BPIV-3)

BPIV-3 is closely related to BRSV and so shares morphological, reproductive and replication strategies [50]. It was found to be one of the most frequently identified viruses amongst recently weaned Irish calves [2].

### 5.5. Bovine Viral Diarrhea Virus (BVDV)

BVDV can cause serious clinical disease in cattle and aid in the development of secondary infections due primarily to its immunosuppressive action [51]. In Irish dairy farms, BVDV was found to have a bulk milk seroprevalence of 88% [43]. A BVDV seroprevalence of 100% was reported in Irish beef herds, with an average within-herd prevalence of 77.7% [45].

### 5.6. Bovine Coronavirus (BCoV)

BCoV can cause respiratory disease in cattle, as well as neonatal calf diarrhoea and winter dysentery in adult cattle [52]. BCoV genome sequences were assembled following a BRD outbreak among Irish beef suckler and pre-weaned dairy calves [53]. BCoV is prevalent amongst cattle herds across a range of countries [54,55]. In Ireland, BCoV was the most frequently identified virus (22.9%) in nasal swabs of calves under 3 months of age [40].

### 5.7. Bovine Adenovirus (BAV)

BAV infections are prevalent in many countries and are associated with enteric and respiratory diseases in cattle [56]. BAV was first identified in the feces of a healthy cow [57] and since then many serotypes have been identified in cattle [58]. Although the pathogenic association of BAV remains uncertain, some serotypes have been isolated in cases of disease. BAV 3 was found to be significantly associated with BRD in California dairy calves with BRD [59]. BAV 6 was identified from a calf that have developed dysentery and died in the UK [60]. In Germany, BAV 7 was isolated from spleen and liver tissue from a deceased newborn Limousin calf [61].

### 5.8. Bovine Rhinitis a Virus 1 (BRAV1) and Bovine Rhinitis a Virus 2 (BRAV2)

Two serotypes have been identified for BRAV (BRAV1 and BRAV2) while BRBV consists of a single serotype [62]. Bovine rhinitis viruses were found to be common in BRD cases in the US with BRAV1, BRAV2 and BRBV co-circulating in US cattle [62].

### 5.9. Influenza C (ICV) and Influenza D (IDV) Virus

Influenza D was identified in Irish bovine samples undergoing routine diagnostics during 2014–2016 [63]. A seroprevalence of 94.6% was found in bovine serum samples taken from healthy cattle pre-slaughter and 64.9% from samples taken from cattle as part of routine BRD diagnostics tests [64]. Higher viral loads of Influenza C and D were detected in US cattle with BRD in comparison to matched controls [65]. Interestingly, most viral infections in BRD calves consisted of a combination of BVDV, IDV and ICV [66].

**Table 1:** An overview of the characteristics, mechanisms of host entry and immunosuppression of common BRD-associated viruses.

Pathogen	Characteristics	Host Entry and Transmission	Mechanism of Immunosuppression/Infection
**Bovine Herpesvirus 1** **(BoHV-1)**	A large, enveloped double-stranded DNA virus of the *Varicellovirus* genus in the subfamily *Alphaherpesvirinae* within the family *Herpesviridae* [67].	Entry through respiratory mucosa; causes epithelial cell apoptosis, Fever, rhinotracheitis, cough, conjunctivitis, oral ulcers; reproductive tract infection with abortion [68].	BoHV-1 can erode mucosal surfaces and cause lesions in the URT [67,69,70]. Acute infection can impair CD8₊ T cell recognition of infected cells and the functioning of CD4₊ T cells [67,71].
**Bovine Respiratory Syncytial Virus** **(BRSV)**	Negative strand RNA virus of the *Paramyxoviridae* family [68].	Entry through respiratory mucosa; Penetrate or degrade the mucous and invade epithelia cells through sialic acid binding [50]. Infects bronchial epithelium, causes syncytial cell formation, bronchiolitis. Fever, cough, increased respiratory rate, depression [68]. Lung pathology as result of BRSV infection is due to the induction of pro-inflammatory cytokines and the inflow of leukocytes, mainly neutrophils [72,73,74].	The attachment protein G aids in the attachment of the virus to host cells and may play a role in immune system interaction [50]. The G protein in BRSV binds to sialic acid residues on cell surfaces within the respiratory tract and with the fusion protein, mediates the attachment and entry of virions to cells [50].
**Bovine Parainfluenza 3** **(BPIV-3)**	An enveloped virus of the *Paramyxoviridae* family containing a non-segmented, single-stranded, negative-sense RNA genome [50].	Transmitted primarily via aerosol through the population [75]. Penetrate or degrade the mucous and invade epithelia cells through sialic acid binding [50]. Shown to infect tracheal cells, ciliated and non-ciliated bronchiolar cells [50] pneumocytes and pulmonary alveolar macrophages [50].	Hemagglutinin-neuraminidase (HN) protein binds to sialic acid residues present on cell surfaces throughout the respiratory tract. The HN proteins with the fusion protein mediate attachment and entry of the virions into the cells [50]. Penetrate or degrade the mucous and infect epithelial cells of the upper respiratory tract through the binding to sialic acid residue (9-carboncarboxylated monosaccharides produced in animals, often used by pathogens as attachment sites [76] on cell membranes [50].
**Bovine Viral Diarrhoea Virus** **(BVDV)**	A positive strand RNA virus of the genus *Pestivirus*, and family *Flaviviridae* [68].	Spread in secretions; causes multiple system disease (abortion, persistent infection) [68]. Infects a wide range of cell types but primarily infects immune cells such as monocytes/macrophages, dendritic cells and lymphocytes [77].	Capable of interfering with type 1 IFN signalling for the establishment of persistent infection [77]. BVDV glycoprotein E^rns^ is able to inhibit IFN expression [78]. The N^pro^ glycoprotein is also essential in the blockage of type 1 IFN [77].
**Bovine Coronavirus (BCoV)**	A single stranded positive-sense RNA virus belonging to the species *Betacoronavirus* 1 (subgenus *Embecovirus*) of the *Betacoronavirus* genus [79,80].	Entry through respiratory mucosa and mouth; infects upper and lower respiratory tract and intestine, causes coughing, fever, rhinitis, loss of appetite, diarrhoea [54].	The HE and S viral proteins aid attachment to N-acetyl-9-O-acetylneuraminic acid to initiate infections [79,81,82,83].
**Influenza D**	A novel RNA pathogen belonging to the family *Orthomyxoviridae.*	Transmission is through direct contact [84] and by aerosol route over short distances; causes lesions in the upper respiratory tract and can replicate in the lower respiratory tract and cause pneumonia [85].	Hemagglutinin-esterase fusion (HEF) glycoproteins aid virus entry to cells [86].
**Influenza C**	An *orthomyxovirus* first detected in BRD cattle in North America in 2016 [87].	Like Influenza D virus, transmission is through direct contact or via aerosol over short distances [88].	The ICV hemagglutinin-esterase (HE) glycoprotein has multiple functions in the viral replication cycle and is the major determinant of antigenicity [89].
**Bovine Adenovirus**	Member of the *adenoviridae* family; non-enveloped double stranded DNA virus [90].	Infections in cattle can be asymptomatic and can occur in the respiratory [91] and alimentary tracts of calves [92].	The viral particle gains entry to host cells by interaction with a primary receptor on the cell surface, followed by interaction with a secondary receptor allowing for viral endocytosis and transportation to the endosome [93,94].

### 5.10. Bacterial Pathogens Associated with BRD

Amongst the most common bacterial pathogens implicated in BRD cases are *Pasteurella multocida, Mannheimia haemolytica, Histophilus somni,* and *M. bovis* [95]. Other bacterial species thought to be involved in BRD include *Mycoplasma bovirhinis* [55,96] and *Moraxella* spp. [97]. *P. multocida* is a potentially zoonotic pathogen shown to be associated with BRD in cattle across the globe and although highly infectious it is not considered highly contagious [98]. *Pasteurellaceae* was amongst the top OTUs (16%) identified in post-mortem lung tissue (16%) and lymph node tissue (8.1%) from Irish dairy calves which died from BRD [99]. *M. haemolytica* was the most frequently identified respiratory pathogen in weanlings (6–12 months of age) submitted to regional veterinary labs in Ireland [2]. *H. somni* was a common pathogen detected in weanlings that died from BRD submitted to veterinary labs in Ireland [2]. *M. bovis* was first identified in Ireland in 1994 [100] and since then has become a major pathogen involved in BRD. A recent study investigating the bulk milk tank seroprevalence of *M. bovis* across farms in Ireland found a herd prevalence of 0.45 [101]. *Mycoplasma bovirhinis* has also been isolated in pneumonic calves [102]. In an US study, *M. bovirhinis* in conjunction with BCoV was found in nursing beef calves during a BRD outbreak [55]. More recently, *M. bovirhinis* was identified as the most frequently identified pathogen from nasal swabs taken from both BRD and asymptomatic dairy calves in southern Brazil [9]. Interestingly, *M. bovirhinis* has also been identified in the nasal bacterial flora of healthy cattle [102] and was increased in the nasal microbiome of healthy Holstein steers (feedlot) cattle relative to cattle with BRD [103]. *Moraxella* spp. has been shown to be involved in keratoconjunctivitis (pinkeye) in cattle [104] and to have a potential role in bovine respiratory health [97]. Furthermore, a recent study examining the effect of respiratory virus vaccination on the bovine respiratory microbiome of feedlot cattle found that cattle that remained healthy had a higher relative abundance of *Moraxella* spp. compared to cattle that developed BRD [105]. An overview of the characteristics and mechanisms of infection of some of the most common bacterial agents implicated in BRD cases is provided in Table 2.

In summary, while BRD is primarily caused by viral and bacterial pathogens, parasitic infections can play a significant role in predisposing cattle to or exacerbating respiratory illness. Among parasites, lungworms, particularly *Dictyocaulus viviparus*, are the most important contributors to respiratory disease in cattle and can complicate the clinical picture of BRD [2,36]. Parasitic infections can exacerbate respiratory conditions by causing immunosuppression, mechanical damage, or chronic inflammation that predispose cattle to secondary infections characteristic of BRD. Although parasites such as lungworms affect the respiratory tract, they are generally considered secondary or complicating factors rather than primary BRD pathogens [2,36].

**Table 2:** An overview of the characteristics, mechanisms of host entry and immunosuppression of common BRD-associated bacteria.

Pathogen	Characteristics	Method of Pathogenesis	Mechanism of Immunosuppression
***Pasteurella multocida***	A small, non-motile, facultative anaerobic, Gram-negative coccobacillus, measuring approximately 0.3 to 1.0 μm in width and 1.0 to 2.0 μm in length. Five capsular serogroups (A, B, D, E, F) [34,106,107] and 16 seroptypes have been classified [34,108].	Commensal bacteria present in the upper respiratory tract; stress resulting from viral infections allow for opportunistic infection of the lung [68].	Virulence factors include a capsule which plays a role in resisting phagocytosis by host cells [34,109]. Outer membrane proteins (e.g., OmpA and type IV fimbriae which may play a role it the adherence to host cells [34,110,111]. Lipopolysaccharide which interacts with the host innate immune response through Toll-like receptors playing a role in the disease process [34,112].
***Histophilus somni***	A non-encapsulated, Gram-negative coccobacillus of the *Pasteurellaceae* family [34].	Commensal bacteria of the upper respiratory tract and reproductive tract, involved in diseases such as thrombotic meningoencephalitis, respiratory disease, myocarditis, polysynovitis, otitis media, mastitis and reproductive tract diseases [34].	Capable of adhering to the endothelial cells causing platelet activation and thrombus formation; the production of lipooligosaccharide can induce apoptosis of endothelial cells [34,113,114]. Capable of biofilm formation with *P. multocida* [34,115].
***Mannheimia haemolytica***	A Gram-negative, non-motile, non-spore forming, facultative, coccobacillus of the *Pasteurellaceae* family [68,116]. Comprises 12 capsular serotypes (A1, A2, A5, A6, A7, A8, A9, A12, A13, A14, A16, A17) [116,117].	Commensal bacteria present in the upper respiratory tract; stress resulting from viral infections allow for opportunistic infection of the lung [68].	Multiple virulence factors including an adhesion, capsular polysaccharide, iron-regulated outer membrane proteins, leukotoxin (Lkt), LPS, lipoproteins, neuraminidase, a serotype-specific antigen, sialoglycoprotease and transferrin-binding proteins [116].
***Mycoplasma bovis***	A wall-less bacterium of class Mollicutes [68].	Causes mastitis, anorexia, nasal discharge; synergistic with other BRD pathogens, forms biofilms to facilitate persistence [68]. Adapted to colonize and persist in mucosal surfaces, with or without causing clinical disease [118].	A lack of a cell wall amongst Mycoplasmas leave membrane proteins exposed and allow them to be the primary interface with the host [118]. Immunodominant variable surface lipoproteins contained by *M. bovis* exhibit strain variation, allowing for a vast antigenic variation in *M. bovis* populations, contributing to immune evasion and persistence [118,119].
***Mycoplasma bovirhinis***	A wall-less bacterium of class Mollicutes [68].	A commonly identified species in respiratory diseases in cattle [120]. Mainly isolated from nasal mucous [121] and lung [55,122].	Adherence proteins allow cell adherence and lipoproteins on the mycoplasma surface modulate interactions between pathogen and host–cell, antigenic variation and immune evasion [120]. Glycerol metabolism and the production of H_2_O_2_ influence *Mycoplasma virulence* [120,123,124].

## 6. Clinical Diagnosis of BRD

**Figure 1:**
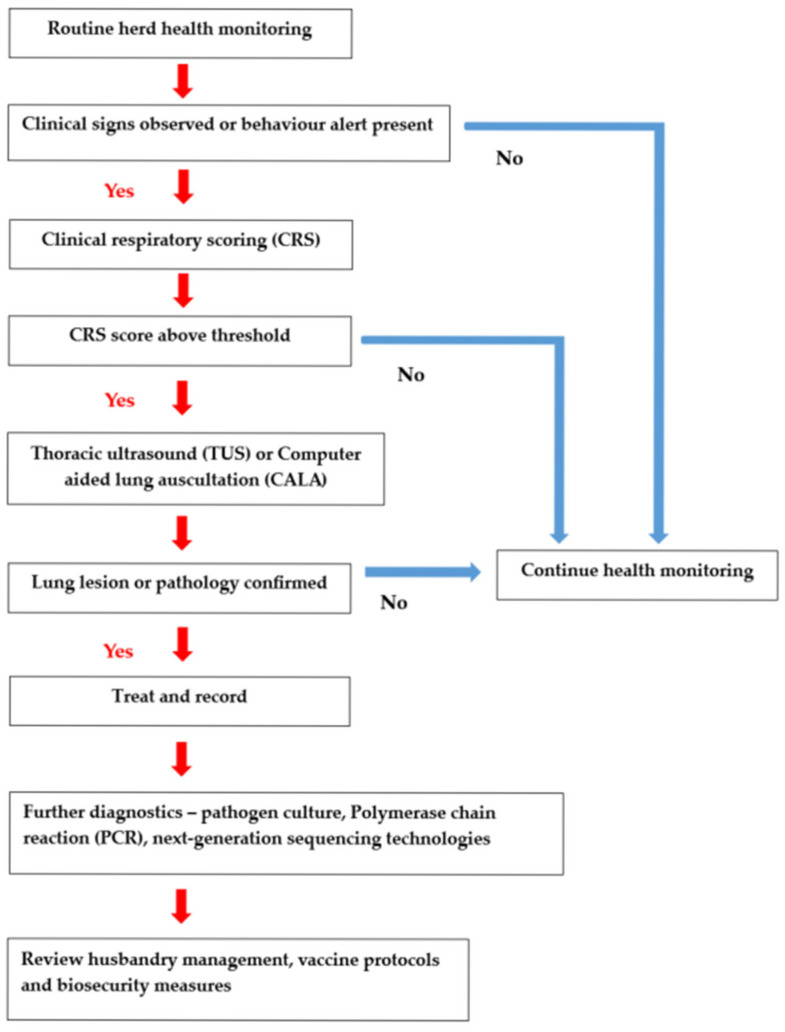
Integrated decision-making flowchart for BRD diagnosis.

A thorough physical examination remains a cornerstone in the clinical diagnosis of BRD. Key examination skills include auscultation of the thorax to detect abnormal lung sounds such as crackles, wheezes, or decreased breath sounds, which may indicate pneumonia or airway obstruction. Measurement of rectal temperature is critical for identifying febrile responses; however, fever patterns can vary depending on the causative pathogen and stage of infection. Assessment of hydration status through evaluation of mucous membrane moisture, skin tenting, and eye recession provides insight into systemic illness and disease severity. Additionally, observation of rumen motility can offer indirect information on the animal’s overall health and response to disease. Respiratory rate and effort, nasal and ocular discharge, coughing, and general demeanour should also be systematically evaluated. Integrating these physical examination findings with clinical respiratory scoring enhances diagnostic accuracy and supports timely intervention. Nonetheless, the accurate and timely diagnosis of BRD remains a considerable challenge within both clinical (veterinary clinics, laboratories) and field (on-farm) settings. Affected animals can display a wide range of symptoms including fever, fatigue, nasal and ocular discharge, coughing and loss of appetite [35]. The severity and duration of these clinical signs can vary depending on the causative pathogen [3]. With disease diagnosis commonly based upon the observation of clinical signs in sick animals, subclinical infections can often go undetected [3]. Accurately diagnosing BRD, especially in distinguishing between viral and bacterial aetiologies and determining whether the infection involves the upper or lower respiratory tract, continues to be a significant challenge [125]. In addition, many of the clinical symptoms are non-specific to BRD meaning a diverse range of pathogens can cause them, thereby making it difficult to pinpoint the exact cause of illness. To address these diagnostic challenges, the subsequent sections will review the clinical diagnostic tools available for BRD, highlighting their applications in identifying the nature of the infection and localizing respiratory tract involvement. An integrated decision-making flowchart for BRD diagnosis is presented in Figure 1. The flowchart shows the sequential application from routine herd health monitoring, clinical scoring, thoracic ultrasound (TUS), and computer-aided lung auscultation (CALA) to guide treatment decisions, pathogen diagnostics, vaccination, protocols and biosecurity measures.

### 6.1. Clinical Respiratory Scoring Methods

BRD diagnostics were, and remain, largely centred on the use of clinical evaluation. The recording of abnormal clinical signs or behaviours is commonly used for the diagnosis of bronchopneumonia and has been used as a “first-line” diagnostic for BRD [125]. These clinical indicators are often assessed using a scoring system, which involves systematically recording each observed sign of disease and assigning corresponding points. The total score reflects the severity of the condition, with higher scores indicating more pronounced clinical signs. Clinical scoring is used in the monitoring and diagnostic of a range of animal health applications across a range of different cohorts [126]. Several systems for assessing clinical respiratory signs of disease in pre-weaned dairy calves use the clinical respiratory score (CRS). These include the Wisconsin calf respiratory score [35], the California score [127] and the Québec modified California score [3]. In addition, a clinical scoring system was developed and validated to enhance the detection and diagnosis of BRD in weaned dairy calves [128]. This system integrates clinical signs including nasal discharge, coughing, eye and ear abnormalities, and rectal temperature, creating a standardized and practical method for early BRD identification. The tool considers various factors such as colostrum management, ventilation, housing conditions, and vaccination protocols [129]. A scoring system serves as a tool that allows scorers, for example, stockperson, technicians, and veterinarians, to implement a structured method for diagnosis for monitoring and assessing animal health. The approaches used are intended to reduce bias and confounding factors and decrease subjectivity. In theory, these systems provide a tool for farmers, technicians, and veterinarians, to monitor the health of livestock and aid in disease diagnosis, whilst decreasing bias and confounding and increase objectivity [130].

Several limitations exist in the use of these scoring systems, with a major issue being their relatively low sensitivity (*Se*) and specificity (*Sp*). The average *Se* of clinical respiratory scores (CRSs) used for detecting BRD in pre-weaned dairy and veal calves was found to range from 30% to 72%, while the *Sp* ranged from 86% to 94% [131]. In practical terms, within a population where BRD prevalence is 20%, this would result in 30% to 70% of affected calves being missed (false negatives), and between 6% and 14% of healthy calves being incorrectly identified as having BRD (false positives), potentially leading to unnecessary treatments [131]. Another major drawback of CRS is that the scorer’s decisions frequently have a significant impact on the outcome. Numerous tests have been found to be very subjective, with results mostly based on the individual conducting the test [16]. Despite training operators or scorers in the use of these systems, agreement between scorers was found to be slight to fair using the 4-scale scoring per clinical sign included in the Wisconsin respiratory scoring chart [125,132]. Clinically, a weak agreement between different operators will not classify a calf similarly [131].

Another limitation surrounding this approach relates to cattle being prey animals by nature, meaning they often hide their clinical symptoms upon observation by a predator, suggesting that some clinical signs of infection may go unrecorded, impacting the resulting score and subsequent diagnosis [125]. The infectious agents involved in BRD often cause a range of non-specific clinical signs such as fever, decreased appetite and depression [95], with other clinical respiratory signs such as increased heart rate, abnormal breathing, nasal and ocular discharge and coughing also commonly observed. Temperature is one of the clinical signs included in these scoring systems, as fever is a non-specific observation of BRD for all major causative agents. However, the length of fever and subsequent decreases and peaks can vary dependent on the pathogen in question, for example, in *M. haemolytica* infections fever is observed the day following challenge with a rapid decrease then seen despite an ongoing infection [95,125]. In viral challenges, such as BoHV-1 infection, rectal temperature was seen to peak at 4 days post infection with a slight decline thereafter [133]. Therefore, the time at which such observations are recorded could influence the results depending on the causative pathogen. While CRS offer a practical and structured approach for early detection of BRD in calves they are limited by low sensitivity, subjectivity, and variability between scorers.

The CRS methodologies described above have been predominantly developed and validated in dairy calf populations, particularly within pre-weaned or recently weaned calves housed in individual hutches or small group pens. These systems may not be directly transferable to beef calves managed in feedlot environments, where group housing, different stressors, and disease dynamics markedly influence clinical presentation. Beef calves in feedlots often exhibit differing behavioural and clinical signs of BRD, such as depression, anorexia, sunken eyes, and drooped ears, which may vary in severity, frequency, and diagnostic relevance compared to dairy calves. Additionally, environmental and social factors in feedlots can affect both the expression of clinical signs and their detection, necessitating tailored scoring protocols. Moreover, differences in pathogen exposure, management practises, and animal temperament contribute to variable disease manifestation. Consequently, clinical scoring systems intended for dairy calves should be applied with caution when assessing beef calves, and ideally, beef-specific scoring criteria should be employed. Recognition of these distinctions is critical to improving the sensitivity, specificity, and overall utility of BRD diagnostic tools across diverse cattle production systems.

### 6.2. Behavioural Monitoring

In addition to the monitoring of clinical signs of disease, the assessment of behaviour is a common method used to assist with the diagnosis of clinical BRD [134]. Behavioural indicators offer a non-invasive and continuous means of monitoring animal health, which is particularly beneficial in large-scale cattle operations where individual clinical assessments are labour-intensive and may delay intervention. Research has shown that pre-weaned dairy calves affected by BRD exhibit significant alterations in daily activity and feeding patterns, including reduced milk and starter intake, increased lying duration, and decreased step counts and activity indices when compared to their healthy counterparts [11]. These changes may reflect underlying discomfort, sickness, or compromised energy balance associated with disease progression. Similar behavioural deviations have been documented in feedlot cattle, where sick animals were observed to spend more time in isolation, modify their feeding bunk attendance across days on feed, and exhibit increased presence near feed and water sources at atypical times of day [135]. Such observations underscore the multifaceted impact of BRD on both social and feeding behaviours and suggest that deviations from established individual baselines may serve as early warning signals of disease onset. A recent study examined how social and movement behaviours in combination with feeding behaviours, can be used with machine learning algorithms to predict BRD in pre-weaned calves [136]. Data were collected from 172 group-housed calves utilizing automatic milk feeding machines and ultra-wideband location sensors. Health evaluations were conducted biweekly employing a modified Wisconsin scoring system, with calves deemed sick if they achieved a Wisconsin score of five or higher and/or exhibited a rectal temperature of 39.5 °C or above. The authors indicated the necessity for further development of the work before behavioural changes can be reliably utilized to predict the onset of BRD in pre-weaned calves [136]. Data from studies such as these relating to behavioural patterns in sick versus healthy cattle may assist in the timely diagnosis of BRD. The incorporation of behavioural data into health monitoring protocols could significantly improve the timeliness and precision of BRD detection, allowing for earlier intervention and more targeted treatments. Ultimately, the integration of behavioural measures with automated monitoring systems may contribute to improved health outcomes, reduced antimicrobial use, and enhanced welfare and productivity in cattle production systems.

### 6.3. Thoracic Auscultation

Thoracic auscultation serves as a valuable tool and is a common step in the assessment of the ruminant respiratory tract [137]. The velocity and turbulence of the air flow in the lungs of healthy animals results in lung sounds being audible and in cases where respiratory infection is present, these sounds become wheezes, because of air turbulence in narrow airways [137]. Crackles, presenting as short popping sounds often occur in cases of bronchopneumonia and result from the sudden opening of obstructed air passages [137]. Although a common approach, limitations exist in using this methodology for BRD diagnosis, as [137] reported only a 5.9% chance of detecting lung consolidation using thoracic auscultation. A more recent development is the use of computer-aided lung auscultation (CALA) for BRD diagnosis. The procedure involves placing the diaphragm of an electronic stethoscope at the fifth intercostal space on the right side of the thoracic wall, approximately 10 cm above the elbow. Lung sounds are recorded continuously for 8 s. The recorded audio signals are then transmitted wirelessly to a nearby computer (within 3 m), where dedicated software performs signal processing. This includes the generation of a spectrogram, removal of heart sounds and environmental noise (such as chute-related sounds), and classification of lung acoustic patterns. The software assigns lung scores on a scale from 1 to 5, representing normal (1), mild acute (2), moderate acute (3), severe acute (4), and chronic (5) respiratory conditions. These lung scores are subsequently relayed back to the stethoscope device and displayed to the user for immediate interpretation [138]. CALA has been used to detect BRD in feedlot cattle with findings showing a substantial agreement (kappa = 0.77) between veterinary auscultation and CALA, and a relatively high *Sp* and *Se* when compared to pen checking [138]. The authors reported that, based on the higher specificity of CALA compared to pen checking, the technology has the potential to reduce the number of cattle falsely diagnosed with BRD, thereby supporting more prudent antimicrobial use in commercial feedlots by minimizing unnecessary treatments. Applying CALA as a follow-up diagnostic tool in cattle initially identified as BRD-affected by pen checking, using a serial interpretation approach assuming conditional independence, can improve the overall specificity of BRD diagnosis in feedlot cattle (combined specificity: Sp_p+CALA_ = Sp_p_ + Sp_CALA_ − (Sp_p_ × Sp_CALA_) = 96.1%) compared to pen checking alone (Spp = 63.0%) [138]. Additionally, the authors suggest that since CALA does not require prior experience in lung auscultation, it can be readily implemented by feedlot personnel responsible for diagnosing and treating BRD [138]. More recently, it was reported that the CALA score collected at the time initial BRD diagnosis and treatment was significantly associated with the risk of BRD retreatment and BRD mortality [139]. Overall, while thoracic auscultation is a commonly used and valuable tool in assessing respiratory health in ruminants, its effectiveness for diagnosing BRD is limited due to its low sensitivity in detecting lung consolidation. CALA offers promising advancement, providing greater diagnostic accuracy and predictive value for BRD outcomes, suggesting its potential as a complementary tool in BRD diagnosis.

### 6.4. Thoracic Ultrasonography (TUS)

In animals affected by BRD, the disease may manifest in a clinical form with obvious signs of infection, or in a sub-clinical form, where typical symptoms are less apparent. In these later instances, the effectiveness of clinical scoring and thoracic auscultation methodologies are limited and can often fail to detect lung lesions. This is evident in recent studies where findings showed that 18.3% (28/153) of purchased suckler bred weanlings [6], and 28.3% (15/53) of purchased dairy-bred calves [7] had lung lesions that were not detected using the Wisconsin calf respiratory scoring chart in the first month after their arrival to a feedlot. To detect these sub-clinical BRD cases, (TUS), which involves a visual inspection of the lung’s appearance in live animals, is often performed. This “silent” presentation of BRD is important since economic losses are associated with sub-clinical BRD [6,7].

Transrectal ultrasound probes, widely used by bovine veterinarians, are suitable for TUS in calves. Handling differs by size: large calves should be restrained in a chute, while smaller calves can be scanned without restraint. Clipping thoracic hair is generally unnecessary but can improve image quality. Isopropyl alcohol (70%) is applied as a coupling agent via spray bottle. Probe depth should be set between 8 and 10 cm for larger calves and 6–8 cm for smaller calves. Both sides of the thorax are examined systematically, with the probe placed in the intercostal spaces parallel to the ribs and moved dorsally to ventrally following hair growth. The scanning sequence starts at the tenth intercostal space and moves cranially to the first intercostal space on the right lung, and from the tenth to the second intercostal space on the left lung. However, access to the cranial thorax in small calves, including the axillary region, is possible with transrectal probes. However, in larger calves restrained in a chute, scanning the first to third intercostal spaces is often not feasible, limiting evaluation of the cranial lung lobes where lesions commonly develop.

TUS has been shown to identify abnormal lung pathologies in calves during experimental challenges with BRSV and *M. haemolytica*, that would not have been identified using clinical scoring alone [140]. A research investigation into the application of TUS for diagnosing and treating BRD on Scottish dairy farms (n = 7) found that of 347 ultrasonographic examinations, 53 (15.3%) were classified as abnormal and 294 (84.7%) as normal. Among 53 classified as abnormal using TUS, only 13 (24.5%) were treated by the farmer; however, of the 294 classified as normal, 22 (7.5%) were treated, indicating that farmers were misdiagnosing BRD in young calves [141].

Unlike clinical respiratory scoring methods, TUS scores have been linked to growth performance in calves with BRD, suggesting that TUS may be a more reliable indicator of the disease’s impact on growth performance. A study examined the impact of clinical BRD and related lung consolidations on the growth performance of recently weaned beef calves during the 65 days after their arrival at the feedlot [6]. Results showed that from day 0 to day 65, ADG did not differ between CRS positive or CRS negative calves. However, calves with positive thoracic ultrasonography findings (TUS+ve) had an ADG that was 0.09 kg/day lower compared to TUS-negative (TUS−ve) calves [6]. Furthermore, calves classified as having BRD (CRS + TUS score ≥ 5) with lung consolidation showed significantly reduced ADG from arrival to day 28 compared to healthy calves and BRD calves without lung consolidation (0.11 ± 0.10 vs. 0.53 ± 0.07 vs. 0.57 ± 0.10 kg/day, respectively); however, no differences in ADG were observed from day 0 to 65 [6]. Using purchased dairy calves, a study examined BRD incidence detected by CRS and/or TUS and the effect of BRD on pre-weaning growth [7]. The authors reported that between purchase (calves; mean age 23 days) and weaning (53 days post-arrival), 43% of calves showed clinical BRD (based on CRS), and 64% had TUS lung lesions [7]. Of the calves with clinical BRD, 61% had lung lesions a median of 10.5 days before clinical signs appeared. Average daily gain was 0.75 kg/day with no difference between calves with or without clinical BRD. However, calves with severe lung lesions gained 0.12 kg/day less than those without lesions [7]. In another study, it was reported that increased TUS scores were linked to reduced ADG in dairy calves [142]. More recent studies [143,144] reported that TUS has demonstrated strong prognostic value, with studies showing that the extent of lung consolidation correlates with higher relapse risk, reduced growth performance, and increased mortality. These findings highlight the value of incorporating TUS with CRS to improve early and accurate detection of clinical and sub-clinical disease in calves. Variation in TUS application highlights the need for system-specific protocols. For example, young dairy calves can be scanned without restraint for thorough lung evaluation, while larger beef calves often require chute restraint, limiting access to some lung areas. This affects lesion detection and clinical decisions, so prevention and treatment strategies should be tailored to each production system for optimal outcomes.

## 7. Vaccination as a Preventive Strategy Against BRD

Variations in vaccine efficacy and optimal vaccination timing between beef and dairy calves are influenced by distinct physiological, immunological, and management factors inherent to each production system. Beef calves are commonly exposed to acute stressors, such as weaning, transportation, and commingling, collectively known as “shipping fever,” which induce transient immunosuppression [4,6,34,42,45]. This immunosuppression heightens susceptibility to bovine respiratory pathogens and can impair the development of protective immunity if vaccination is not administered sufficiently prior to these stress events. Therefore, timely vaccination and appropriate booster administration before feedlot entry is critical for achieving effective immunoprotection in beef calves. In contrast, dairy calves typically develop enzootic pneumonia during the pre-weaning to early post-weaning period (2–6 months of age), often within group housing systems characterized by continuous exposure to respiratory pathogens [3,7,11,12,28,142]. At this stage, maternally derived antibodies (MDA) are generally present at high titers and may interfere with vaccine antigen recognition, attenuating the calves’ active immune responses, particularly to inactivated vaccines. Furthermore, the temporal pattern of BRD incidence in dairy calves is less associated with discrete stressors compared to beef calves, complicating the identification of an optimal vaccination window. Recognizing these mechanistic distinctions is essential for the development of tailored vaccination protocols that maximize protective efficacy against BRD within specific cattle production systems.

Antimicrobial metaphylaxis has historically been employed as a preventative strategy to reduce the incidence of BRD development in high-risk cattle populations. However, increasing regulatory restrictions on antimicrobial use, coupled with concerns regarding the contribution of metaphylaxis to AMR, have prompted a re-evaluation of this approach. In the context of BRD, the reliance on antibiotics is particularly critical, as they remain a primary treatment option for secondary bacterial infections, especially in severe disease cases. Consequently, emphasis has shifted toward the implementation of comprehensive disease prevention strategies that prioritize good husbandry and management practises, alongside effective vaccination programmes. These integrated approaches aim to reduce disease incidence, improve animal welfare, and mitigate the risk of AMR development. Although vaccines are currently used to prevent BRD outbreaks, the disease continues to be a significant challenge in dairy and beef production systems globally [6,28,29,145,146].

There are many types of vaccines used against both viral and bacterial BRD pathogens in both beef and dairy cattle. Viral vaccines either contain modified-live virus (MLV) or inactivated virus [147,148,149]. Multivalent viral vaccines, targeting more than one virus, are commercially available in Ireland. Generally, MLV vaccines have been shown to induce strong humoral and cell-mediated immunity with fewer doses required to provide protection [150,151,152]. Killed or inactivated viral vaccines induce robust humoral responses but weaker cell-mediated immunity and require at least two doses to provide adequate protection [153]. Additionally, bacterin and/or leukotoxoid vaccines are available against bacterial BRD pathogens such as *M. haemolytica*, *P. multocida* and *H. somni* [154].

The effectiveness of BRD vaccines for disease prevention has been the subject of many studies. The duration of immunity provided by a quadrivalent vaccine targeting BoHV-1, PI3, BVDV, and BRSV was studied, with results indicating reduced viral shedding in vaccinated animals compared to controls [155]. Beef steers that received a modified live vaccine (MLV) against BVDV demonstrated improved feed intake and feeding behaviours after viral challenge compared to those given either a killed vaccine or no vaccine [156]. Additionally, vaccination with a quadrivalent vaccine (inactivated BoHV-1, MLV BRSV, inactivated BVDV, and MLV PI3) followed by a booster 21–27 days later significantly decreased morbidity and mortality rates from BRD in vaccinated animals compared to unvaccinated controls [157]. A recent study reported a BRSV outbreak on a single farm where vaccinated animals showed significantly reduced morbidity (20.4%) and disease severity (score of 1.70) compared to unvaccinated controls, which had morbidity of 53.7% and a severity score of 2.11 [158]. Overall, vaccinated animals had a significantly lower number of cases (0.36 vs. 0.64 cases/calf), lower morbidity (26.78% vs. 41.24%), and lower antimicrobial treatments (33.3% vs. 57.4%). In addition, vaccinated animals exhibited a numerically higher average daily weight gain (ADWG) and a significantly greater carcass weight compared to controls, with increases of 35.78 g/day and 6.58 kg, respectively. However, vaccination against BRD is not always successful and certain factors can influence the efficacy of vaccines. Calves which had pre-existing maternally derived antibodies (MDAs) were reported to respond poorly to foot and mouth disease vaccination [159]. The administration of an intramuscular MLV vaccine against BVDV 1 and 2, BRSV, BoHV-1 and PI3 to dairy heifer calves found no difference in the risk of developing BRD or the risk of mortality between vaccinated and unvaccinated calves, with MDA interference suggested as a possible reason for these findings [160]. In a study comparing the efficacy of a bivalent modified-live (ML) vaccine against BRSV and PI3 virus in Czech-pied Holstein calves with and without MDAs, vaccinated calves showed significantly lower clinical scores and reduced viral nasal shedding compared to unvaccinated controls, regardless of the presence of MDAs [161]. Furthermore, it was demonstrated that a live intranasal vaccine triggered an anamnestic IgG response after a BRSV challenge in calves regardless of the presence of MDAs [162]. This indicates that early intranasal vaccination may be an effective approach to protect calves during the critical period when MDA levels are declining but active immunity has not yet been fully established [162].

BRD pathogenesis often differs between beef and dairy cattle, which can impact vaccine effectiveness. Dairy calves often develop enzootic pneumonia between 2 and 6 months of age, while beef calves typically experience ‘shipping fever’ after weaning, often due to stress [2]. These differences affect the timing of peak BRD incidence, vaccination schedules, and the influence of maternal antibodies. It was suggested that these factors likely played a role in the significantly lower morbidity and mortality seen in vaccinated beef calves, a benefit that was not observed in vaccinated dairy calves compared to unvaccinated controls during natural exposure trials [147]. Additionally, the timing of vaccination is a crucial element influencing vaccine efficacy. Vaccinating feedlot cattle 15 days before weaning, followed by a booster 15 days before entering the feedlot, led to higher serum antibody titers against BRD viruses (BVDV, BoHV-1, BRSV, and PI3) at feedlot entry and a decreased incidence of BRD during the feedlot period compared to both control and delayed vaccination groups [163]. Recently, a study investigating the timing of administering a polyvalent (BoHV-1, PI3, BVDV-1, BVDV-2 and BRSV) vaccine found that animals that received a vaccination upon feedlot arrival had significantly lower antimicrobial use, greater average daily gain, and reached slaughter age earlier than animals that were vaccinated later [164]. While challenge studies have proven effective in evaluating vaccine efficacy, they are typically carried out under highly controlled conditions, which differ significantly from the more variable environments found on farms [165]. Additionally, the timing of viral challenge may not always concur with the timing of viral/bacterial outbreaks in a natural setting [149]. Therefore, further research on the efficacy of vaccines during natural field infections is warranted. Although evidence shows that many vaccines are effective in reducing morbidity and mortality in BRD cases, disease burden remains high in cattle populations and so further preventative measures are required to lower the incidence of BRD.

Recent studies have identified emerging pathogens, such as Influenza D virus, as contributing agents in the complex etiology of BRD. Currently, these pathogens are not incorporated into existing vaccination programmes, which primarily target well-established viral and bacterial agents. The role of emerging pathogens in BRD pathogenesis remains incompletely understood, and their inclusion in vaccination protocols has not yet been realized. This represents a critical research gap that warrants further investigation to elucidate their epidemiology, pathogenicity, and potential impact on vaccine efficacy. Developing vaccines targeting these novel agents could enhance disease control strategies and reduce the overall burden of BRD in cattle populations.

## 8. Prevention of BRD

Effective prevention of BRD requires a multifaceted approach centred on reducing stress [166], implementing timely vaccination protocols, and managing concurrent health burdens such as parasitism [167,168]. In the past, prophylactic antimicrobial treatments were commonly used in high-risk weanlings to mitigate the effects of pneumonia, often compensating for suboptimal management. However, with the implementation of EU Regulation 2019/6 [169], the routine preventive use of antimicrobials in healthy animals has been banned. As a result, meticulous planning and management around the weaning period have become essential to BRD control. While vaccination remains a critical component in enhancing immunity against key BRD pathogens, it is not a substitute for sub-optimal husbandry management practises. Optimal nutrition, appropriate housing and ventilation, stress reduction, good Stockpersonship, and consideration of environmental factors such as weather and seasonal changes are all essential elements that influence BRD risk and animal resilience. Good husbandry practises including, ensuring adequate colostrum intake [170], minimizing transport stress [171,172], and maintaining clean, well-ventilated housing environments [173], help reduce the incidence and severity of BRD.

In the context of BRD prevention, the role of stress as a predisposing factor cannot be overstated. Stress disrupts normal immune function, rendering calves more vulnerable to respiratory pathogens [174]. While the physiological and psychological stresses of weaning are well-documented [175,176,177,178], it is equally important to consider the compounding impact of husbandry procedures such as dehorning [179] and castration [180] when these are carried out in close temporal proximity to weaning [2]. Both dehorning and castration are known to induce acute stress responses in calves, including elevated cortisol levels, behavioural distress, and inflammation [179,180]. These responses may compromise immune function, creating an immunosuppressive window during which the risk of BRD increases significantly. When these procedures coincide with other stressors, such as weaning, housing, or transport, the cumulative burden can overwhelm the calf’s ability to mount an effective defence against respiratory pathogens [6,7,28,34,142]. To mitigate this risk, best practise guidelines recommend performing castration and dehorning at least four weeks prior to weaning, preferably during early life stages when the stress response and risk of complications are reduced [179,180]. Integrating the timing of these interventions into a comprehensive weaning management plan is therefore essential. This not only improves calf welfare but also supports the effectiveness of other BRD prevention strategies, such as vaccination and nutritional support. Additionally, monitoring animal behaviour and early clinical signs supports timely intervention, which is key to effective disease control [181].

Although this review focuses primarily on clinical diagnostic methods and vaccination strategies, we emphasize that these should be implemented within an integrated management framework. Addressing underlying management and environmental factors is fundamental to successful BRD prevention and to reducing reliance on antimicrobial treatments. Future reviews could explore in greater depth the roles of nutrition, housing design and air microbiome, Stockperson training, environmental management in BRD control, as these factors are critical in supporting overall herd health and welfare and reducing AMR development.

## 9. Conclusions

BRD remains a critical health challenge in cattle worldwide, significantly impacting animal welfare and industry profitability. Key viruses such as BoHV-1, BRSV, BPIV-3, BVDV, and BCoV are highly prevalent and play significant roles in the onset and progression of BRD. Emerging viruses like Influenza D and C are increasingly recognized as important contributors. The endemic nature of these viruses, especially BoHV-1 and BVDV, alongside their capacity for latency and immunosuppression, complicates disease control.

BRD is also associated with a diverse range of bacterial pathogens, including *Pasteurella multocida*, *Mannheimia haemolytica*, *Histophilus somni*, *M. bovis*, and *Mycoplasma bovirhinis*, each exhibiting varying roles in disease progression and prevalence across regions. Emerging evidence highlights the complexity of the bovine respiratory microbiome, where opportunistic pathogens such as *Moraxella* spp. and *M. bovirhinis* may also contribute to respiratory health or disease, depending on host and environmental factors. The complexity of the multifactorial causes of BRD and the frequent presence of subclinical infections complicate timely and accurate diagnosis. While traditional clinical scoring methods provide a useful framework, their limitations in sensitivity and subjectivity highlight the need for more objective diagnostic tools. Advances such as TUS and behavioural monitoring show promise in improving early detection and disease management. Addressing BRD effectively requires integrated approaches combining improved diagnostics, targeted treatments, vaccination and preventive strategies to reduce antimicrobial use and mitigate economic losses. Continued research and innovation in diagnostics and management practises are essential to control BRD and support sustainable cattle production.

## 10. Implications

The challenges posed by BRD have significant implications for animal health, farm economics, and public health. Inaccurate or delayed diagnosis can lead to untreated or improperly managed infections, increasing mortality rates and reducing growth performance in affected cattle. This results in direct financial losses for farmers due to decreased productivity and increased treatment costs. Moreover, reliance on antimicrobials for BRD treatment raises concerns about AMR, which threatens both animal and human health globally. In summary, the implications of BRD extend beyond immediate animal health issues, influencing economic sustainability, public health, and animal welfare, underscoring the urgent need for improved diagnostic and preventive strategies.

## 11. Future Work

Effective control of BRD requires an integrated, multifactorial approach. While current strategies have improved early detection and mitigation, several areas warrant further investigation to optimize long-term outcomes.

Diagnostic tools including TUS, CALA, and CRS require validation across diverse production systems. Research should focus on tailoring these tools to different animal populations (dairy and beef), housing types, animal ages, and management conditions to ensure diagnostic accuracy and consistency. For example, TUS protocols may vary depending on whether calves are restrained or not, and CRS tools validated in dairy systems often lack sensitivity and specificity when applied to feedlot beef calves. Future work should focus on tailoring and validating these diagnostic protocols to the unique handling and environmental conditions found in beef feedlots, dairy farms, and veal production systems.

Automated behavioural monitoring, remote temperature sensing, and AI-driven health alert systems offer promise for real-time BRD detection. These technologies require validation across commercial settings and integration with decision-support frameworks to enable early, targeted interventions.

Environmental conditions—including ventilation, dust levels, humidity, ammonia, and other noxious gasses—substantially impact respiratory health. In addition to these abiotic factors, the air microbiome is increasingly recognized as a potential contributor to BRD risk, influencing pathogen exposure and immune responses. Future studies should characterize airborne microbial communities in cattle housing environments and assess their association with BRD incidence.

Emerging pathogens such as IDV are increasingly implicated in BRD but remain unaddressed by current vaccines. Future research should prioritize defining the epidemiology, virulence, and interactions of pathogens with co-infecting agents to inform the development of multivalent or platform-based vaccines. Treatment approaches, especially those integrated with advances in molecular and laboratory diagnostics of BRD pathogens, are beyond the scope of this review. These interventions, along with improved molecular diagnostics, hold significant potential to reduce antimicrobial use—a critical step in combating AMR.

The substantial economic burden of BRD highlights the urgent need for improved prevention and control strategies, particularly in the context of vaccination. Variability in vaccine efficacy, age-dependent responses contribute not only to ongoing treatment costs but also to long-term productivity losses. Integrating these challenges into economic modelling frameworks will provide a more accurate assessment of cost-effectiveness, thereby informing optimized vaccination protocols and resource allocation strategies tailored to specific production systems.

## Data Availability

Data are contained within the review paper.

## Abbreviations

The following abbreviations are used in this manuscript:

ADG	Average daily gain
AMR	Antimicrobial resistance
BCoV	Bovine coronavirus
BoHV-1	Bovine herpesvirus 1
BPI3V	Bovine parainfluenza 3
BRAV1	Bovine rhinitis A virus 1
BRAV2	Bovine rhinitis A virus 2
BRD	Bovine respiratory disease
BRSV	Bovine respiratory syncytial virus
BVDV	Bovine viral diarrhoea virus
CALA	Computer Aided Lung Auscultation
CRS	Clinical respiratory scoring
HN	Hemagglutinin-neuraminidase
IDV	Influenza D virus
IFN	Interferon
*Se*	Sensitivity
*Sp*	Specificity
Lkt	Leukotoxin
LPS	Lipopolysaccharide
LRT	Lower respiratory tract
MLV	Modified live vaccine
*M. bovis*	*Mycoplasma bovis*
TUS	Thoracic ultrasonography
US	Unites states
URT	Upper respiratory tract
WBC	White blood cell
